# Association between oropharyngeal ph-monitoring, pepsin saliva concentration and degree of apnea–hypopnea index of obstructive sleep apnea

**DOI:** 10.1186/s40463-023-00675-0

**Published:** 2023-10-14

**Authors:** Francois Bobin, Jérôme R. Lechien

**Affiliations:** 1 Department of Otolaryngology, Polyclinic of Poitiers, Elsan Hospital, Poitiers, France; 2 Department of Human Anatomy and Experimental Oncology, Faculty of Medicine, UMONS Research Institute for Health Sciences and Technology, University of Mons (UMons), Mons, Belgium; 3 Department of Otolaryngology-Head & Neck Surgery, Laryngoloy and Bronchoesophagology Division, EpiCURA Hospital, University of Mons, Mons, Belgium; 4 Department of Otolaryngology, Foch Hospital, Paris Saclay University, Suresnes, France

**Keywords:** Laryngopharyngeal, Reflux, Otolaryngology, pH monitoring, Pepsin, Sleep, Apnea, Laryngeal, Voice, Head neck, Laryngology

## Abstract

**Objective:**

To investigate the association between obstructive sleep apnea (OSA) and laryngopharyngeal reflux (LPR) through oropharyngeal pH-monitoring and pepsin saliva measurements.

**Design:**

Prospective uncontrolled study.

**Methods:**

Patients with sleep disturbances and reflux symptoms underwent polysomnography, 24-h oropharyngeal pH-monitoring and saliva pepsin collections. The prevalence of LPR was investigated in OSA patients according to oropharyngeal pH-monitoring and pepsin measurements. A correlation analysis was performed between pH-monitoring findings, pepsin saliva levels, reflux symptom score-12 (RSS-12), reflux sign assessment (RSA), Apnea–Hypopnea Index (AHI), Epworth Sleepiness Scale, Pichot and arousal findings.

**Results:**

Thirty-seven patients completed the evaluations. LPR was detected in 34/37 (92%) and 29/34 (85%) patients at the oropharyngeal-pH monitoring and pepsin test, respectively. OSA was detected in 30 patients (81%). Among them, LPR was detected in 28/30 (93%) cases. Pharyngeal reflux events mainly occurred nighttime/supine in OSA patients. Both Ryan score and supine reflux time at pH < 6.5 were significantly associated with BMI and the RSA sub- and total scores (*p* < 0.02). Tongue-base hypertrophy score was positively associated with the number of micro-arousals (*p* = 0.027); the supine percent of pH < 6.5 (*p* = 0.030); morning (*p* = 0.030) and bedtime pepsin saliva measurements (*p* = 0.037). The bedtime pepsin saliva level was significantly associated with Ryan Score (*p* = 0.047); AHI (*p* = 0.017) and the sleep saturation < 90% time (*p* = 0.040). The saliva level of the morning pepsin was associated with a shortest paradoxical sleep phase (*p* = 0.013).

**Conclusion:**

OSA patients may have high prevalence of pharyngeal reflux events at the oropharyngeal pH-monitoring and high pepsin saliva measurements. Oropharyngeal pH-monitoring should be useful for the correlation between reflux and sleep findings in OSA patients. Future large cohort controlled studies are needed to determine the prevalence of LPR in OSA and healthy individuals.

## Introduction

Laryngopharyngeal reflux (LPR) is an inflammatory condition of the upper aerodigestive tract tissues related to direct and indirect effect of gastroduodenal content reflux, which induces morphological changes in the upper aerodigestive tract [1]. Because the deposit of pepsin induces inflammatory reaction in mucosa, reflux was suspected to be associated with many common inflammatory otolaryngological conditions, including Eustachian tube dysfunction and related otitis media [2], chronic rhinosinusitis [3], subglottic stenosis [4], tobacco-induced laryngopharyngitis [5], and nonfunctional laryngeal disorders [6]. An increasing number of studies reported the coexistence between LPR and obstructive sleep apnea (OSA) but the exact prevalence of pharyngeal reflux events, the reflux profile of OSA patients and the potential association with sleep parameters remain unclear [7–9]. Precisely, a few studies investigated the occurrence of LPR in OSA patients through objective diagnostic tools such as hypopharyngeal-esophageal multichannel intraluminal impedance-pH testing or oropharyngeal pH-monitoring [7]. However, the identification of LPR-OSA coexistence appears important to improve the management of patients. Indeed, some studies reported that OSA patients may have worse symptoms of LPR [9], while others showed a benefit of antireflux therapy in OSA patients, which is associated with an improvement in daytime sleepiness and a reduction in nocturnal reflux-related arousals [8].

The aim of this study was to investigate the association between obstructive sleep apnea syndrome and laryngopharyngeal reflux in OSA patients through oropharyngeal pH-monitoring and pepsin saliva detection.

## Methods

Forty-five adult patients with primary patient-reported sleep disturbances (fatigue, snoring) and LPR symptoms and findings [10] were prospectively recruited from April 2021 to March 2022 in Elsan Medical Center (Poitiers, France). The prevalence of LPR and OSA was assessed in patients through objective diagnostic tools. The LPR diagnosis consisted of > 1 pharyngeal reflux events at the oropharyngeal pH-testing. The diagnostic of OSA was considered according to the polysomnography (apnea–hypopnea index (AHI) ≥ 5) [11], while the LPR diagnosis was carried out with 24-h oropharyngeal pH-monitoring. The included patients underwent simultaneously 24-h oropharyngeal pH-monitoring and polysomnography (Cidelec LXe, Loire, France). Moreover, the saliva of patients was collected the day of the pH-testing in the morning (fasting) and at bedtime. Gastrointestinal (GI) endoscopy was proposed to patients with GERD-related symptoms and in elderly patients (> 55 years old) who are known to less feel GERD-symptoms [12]. The following exclusion criteria were considered: smoker, alcohol dependence, previous OSA antireflux therapy, neurological or psychiatric illness, head and neck malignancy, history of head and neck radiotherapy, history of gastroesophageal or upper digestive surgery, active seasonal allergies, asthma and inhaled corticosteroid use. The Elsan Ethics committee approved the study protocol (PPC France Nord-West, n°2020-A02789-30). The informed consent was obtained for all patients.

### Clinical evaluations

The symptoms of reflux were evaluated with the Reflux Symptom Score-12 (RSS-12), which is a validated 12-item patient-reported outcome assessing symptom frequency, severity and their impact on patient quality-of-life. RSS-12 score > 11 is suggestive of LPR, exhibiting a sensitivity of 94.5% and a specificity of 86.2%.^10^ Reflux signs were evaluated with the Reflux Sign Assessment, which is a 61-point validated clinical instrument that assessed the presence and severity of oral, pharyngeal and laryngeal findings [13].

### Oropharyngeal pH-monitoring

Patients with RSS-12 > 11 underwent 24-h oropharyngeal pH-monitoring (Restech Dx‐pH, Restech, San Diego, CA). The probe was placed in the oropharynx of fasting patient at 9:00 A.M. through a standardized and recommended method by the provider. According to a recent meta-analysis, the LPR diagnostic was based on the presence of > 1 pharyngeal reflux event [14]. The following data were collected from the analysis: total number of reflux event lasting > 5 min; Ryan score; pH total time below/at pH 6.5., 6.0, 5.5 and 5.0 in both upright and supine position.

### Pepsin saliva measurement

Simultaneously to oropharyngeal pH-monitoring, the saliva of patients was collected twice: in the morning (fasting) and at bedtime. The first saliva sample was collected in the evening at the bedtime (the day before the removal of the pH testing probe). The second sample consisted of the morning saliva before the removal of the pH study probe. These both pepsin measurements allowed the investigation of association between saliva pepsin concentration and the occurrence of pharyngeal reflux events daytime and nighttime, simultaneously to the polysomnography. The saliva samples (Peptest®; RDBiomed Ltd., Hull, United Kingdom) were collected and the measurement of pepsin concentration was performed by a trained lab technician according to a standardized procedure reported in previous studies [15, 16]. The saliva pepsin concentration was measured with the Cube Reader® that detects pepsin down to 16 ng/mL. The test was considered as positive when the pepsin level reached ≥ 16 ng/mL [16].

### Sleep findings

Polysomnography (PSG) was carried out at home during the 24-h pH-monitoring period with the Cidelec CID-LXe (Cidelec, Loire, France) that is composed of 20 channels. Patients fulfilled the French version of Epworth Sleepiness Scale (ESS) [17] and the Pichot fatigue scale [18]. The following PSG data were extracted by a board-certified sleep physician using the Cidelec software (Cidelec v2.2.6., Loire, France): Apnea–Hypopnea Index (AHI; Chicago scoring system) [11]; total number of arousals; number of arousals/hour; % of time with O_2_ saturation level < 90%; and the 4 sleep phases including the paradoxical sleep. OSA status was retrieved from the home sleep study findings considering an AHI ≥ 5 per hour as a positive OSA diagnosis. The degree of obstructive sleep apnea was rated regarding the report of the American Academy of Sleep Medicine based on the patient’s AHI: mild (5–14 events/hour); moderate (15–30 events/hour); or severe (> 30 events/hour) [11].

## Statistical methods

Statistical analyses were performed wirh the Statistical Package for the Social Sciences for Windows (SPSS version 22,0; IBM Corp, Armonk, NY, USA). The relationships between pH study findings, pepsin saliva concentration, symptoms, signs and sleep findings were investigated through correlation analysis. A level of significance of *p* < 0.05 was used.

## Results

**Fig. 1:**
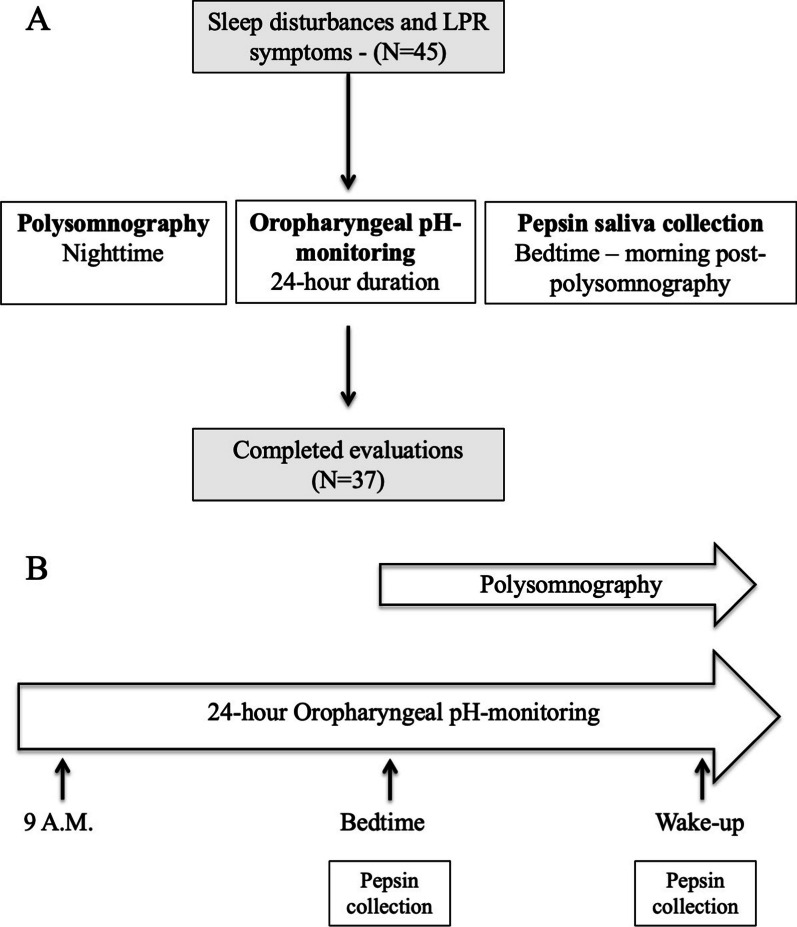
Chart flow. The figure represents the flow chart (**A**) and the performed evaluations over the 24-h testing period (**B**)

Thirty-seven adult patients met the inclusion criteria and completed the evaluations (Fig. 1). The mean age of patients was 51.2 ± 13.5 years old. The mean body mass index (BMI) was 29.5 ± 6.1, ranging from 20 to 42 (Table 1). OSA was detected in 30 patients (81%), corresponding to 10, 12, and 8 mild, moderate and severe OSAS, respectively. LPR diagnostic was found at the oropharyngeal pH study in 34 patients (92%). Among patients with a contributive pepsin test, 29/34 (85%) reported a positive morning or bedtime pepsin test. The level of saliva pepsin was not available in 3 patients because the pepsin tests were not interpreted due to sticky saliva. One patient had only one readable pepsin test. The gastrointestinal endoscopy was performed in 16/37 patients (Table 1). Oropharyngeal pH-monitoring findings are available in Table 1. The reflux symptoms and findings of patients are described in Table 2. The mean RSS-12 was 60.6 ± 47.1. The mean RSA was 20.7 ± 7.8 (Table 2). The sleep features are reported in Table 3. The mean AHI was 19.9 ± 19.0 and ranged from 0 to 78. Patients reported a mean Epworth score of 8.3 ± 5.8, whereas the Pichot score was 11.1 ± 8.9. The data of sleep phases N1, N2, N3, and paradoxical sleep are reported in Table 3. The mean total number of arousals was 151.8 ± 129.4.

**Table 1:** Epidemiological and clinical features of patients

Clinical features		Range
Age	51.2 ± 13.5	18–72
BMI	29.5 ± 6.1	20–42
Gender (F/M)	17/20	–
*Gastrointestinal endoscopy*	N = 16	
Normal	1 (6)	–
Esophagitis	2 (13)	–
Hiatal hernia	14 (88)	–
LES insufficiency	15 (94)	–
Gastritis	0 (0)	–
*Oropharyngeal pH study (m ± SD)*		
Ryan score (N < 6.8)	7.2 ± 9.0	
Number of events pH < 6.5*	13.5 ± 7.3	0–33
Supine time of events pH < 6.5	71.4 ± 32.5	0–100
Upright time of events pH < 6.5	48.4 ± 28.8	0.86.8
Number of events pH < 6.0	11.0 ± 7.9	0–27
Supine time of events pH < 6.0	48.2 ± 35.0	0–97.8
Upright time of events pH < 6.0	18.3 ± 17.6	0–52.0
Number of events pH < 5.5	5.6 ± 6.1	0–18
Supine time of events pH < 5.5	23.0 ± 25.8	0–89.6
Upright time of events pH < 5.5	4.3 ± 6.4	0–22.1
Number of events pH < 5.0	2.2 ± 3.0	0–9
Supine time of events pH < 5.0	8.5 ± 13.5	0–64.2
Upright time of events pH < 5.0	1.2 ± 2.0	0–7.5
Pepsin findings		
Pepsin saliva concentrations (ng/mL)		
Morning (m ± SD)	61.1 ± 80.5	0–302
Evening (m ± SD)	82.4 ± 95.3	0–375
Abnormal pepsin levels		
Morning	14 (38)	–
Evening	20 (54)	–
At least one abnormal measure	27 (73)	–

*Number of reflux event lasting > 5 min

*BMI* body mass index, *m* mean, *N* number of patients, *SD* standard deviation

**Table 2:** Symptoms and findings of patients

RSS-12 outcomes	Mean SD
Voice disorder	1.6 ± 3.5
Throat pain or odynophagia	1.2 ± 2.5
Dysphagia	1.8 ± 4.2
Throat clearing	4.1 ± 5.8
Globus sensation	3.3 ± 5.7
Excess throat mucus	6.5 ± 8.4
Halitosis	5.4 ± 8.0
Heartburn, stomach acid coming up, regurgitations, burps, nausea	10.6 ± 9.8
Abdominal pain or diarrheas	6.0 ± 7.4
Indigesiton, abdominal distension and/or flatus	9.1 ± 9.2
Cough after eating or lying down or daytime troublesome cough	4.4 ± 5.5
Breathing difficulties, breathlessness, or wheezing	6.5 ± 7.4
*Quality of Life score*	22.2 ± 23.4
*RSS-12 total score*	60.6 ± 47.1
*RSA outcomes*	
Anterior pillar erythema	2.5 ± 2.0
Uvula erythema ± edema	0.7 ± 1.3
Coated tongue	1.9 ± 0.5
*Oral cavity subscore*	5.2 ± 2.0
Posterior oro- or hypopharyngeal wall erythema	3.4 ± 1.4
Posterior oro- or hypopharyngeal wall inflammatory granulations	0.1 ± 0.5
Tongue tonsil hypertrophy	1.4 ± 1.7
Contact between epiglotitis and tongue tonsils	1.1 ± 1.8
Pharyngeal sticky mucus	2.4 ± 2.0
*Pharyngeal cavity subscore*	*8.8* ± *3.8*
Ventricular band erythema ± edema	0.1 ± 0.1
Epiglottis erythema	0.8 ± 1.4
Commissure posterior/arytenoid erythema	3.2 ± 2.0
Inter-arytenoid granulatory tissue	0.1 ± 0.1
Posterior commissure hypertrophy	1.9 ± 2.4
Retro-cricoid erythema	0.5 ± 1.1
Retro-cricoid edema	0.2 ± 0.9
Endolaryngeal sticky mucus deposit	0.3 ± 0.9
*Laryngeal subscore*	7.0 ± 3.8
*RSA Total score*	20.7 ± 7.8

*RSA* reflux sign assessment, *RSS-12* reflux symptom score-12, *SD* standard deviation

**Table 3:** Sleep findings

Sleep features	Mean, SD	Range
Apnea–Hypopnea Index	19.9 ± 19.0	0–78
No OSAS	7 (19)	–
Mild OSAS	10 (27)	–
Moderate OSAS	12 (32)	–
Severe OSAS	8 (22)	–
Sleep phases (min)		
Phase 1	49.8 ± 49.0	6–263
Phase 2	273.5 ± 72.2	93–333
Phase 3	85.9 ± 35.9	32–160
Paradoxical sleep	76.4 ± 34.7	8–143
Arousal data		
Arousal (tot N)	151.8 ± 129.4	24–411
Arousal (N/hour)	15.4 ± 17.1	1–47
Episodes of saturation < 90%	6.4 ± 17.2	0–94
Epworth Sleepiness Scale	8.3 ± 5.8	0–29
Pichot Score	11.1 ± 8.9	0–32

*OSAS* obstructive sleep apnea syndrome, *SD* standard deviation

### Prevalence of reflux

The reflux was diagnosed in 34/37 patients with sleep disturbances and LPR-symptoms (97%). Among patients with a positive LPR diagnosis at the oropharyngeal pH-monitoring and contributive pepsin test, 27/31 (87%) had at least one positive pepsin test. The three patients without LPR at the oropharyngeal pH-monitoring had at least one positive pepsin measurement.

Among OSA patients, 28/30 (93%) had positive LPR at the oropharyngeal pH-monitoring. Peptest was positive in 22/27 OSA patients (81%) with a positive LPR diagnosis at the oropharyngeal pH-monitoring.

### Associations

The association analysis reported significant positive associations between the BMI and the following sleep and reflux parameters: total number of arousals (r_s_ = 0.392; *p* = 0.015); RSA (r_s_ = 0.587; *p* < 0.001); reflux time at pH < 6.5 (r_s_ = 0.447; *p* = 0.004) and Ryan score (r_s_ = 0.385; *p* = 0.015). There were significant positive associations between Ryan score and the following: level of bedtime pepsin (r_s_ = 0.338; *p* = 0.047); oral (r_s_ = 0.380; *p* = 0.017), pharyngeal (r_s_ = 0.554; *p* < 0.001), laryngeal signs (r_s_ = 0.378; *p* = 0.041), total RSA (r_s_ = 0.539; *p* < 0.001), meaning that patients with highest Ryan score reported highest RSA sub- and total scores. Similar observations were found for reflux time at pH < 6.5 in supine or upright position (Table 4).

**Table 4:** Associations between reflux and sleep outcomes

		Reflux outcomes											
		Morning	Bedtime	Oral	Pharynx	Tonsil	Larynx	Total	Total	Ryan	pH < 6.5	pH < 6.5	pH < 6.5
Reflux outcomes	BMI	pepsin	pepsin	RSA	RSA	Score	RSA	RSA	RSS − 12	Score	Supine	Upright	N (tot)
Ryan score	0.385*	0.163	0.338*	0.380*	0.554***	0.382*	0.378*	0.539***	− 0.039	–	–	–	–
Reflux pH < 6.5 supine	0.447**	0.226	0.181	0.109	0.625***	0.358*	0.525***	0.678***	0.129	–	–	–	–
Reflux pH < 6.5 upright	0.132	0.147	0.064	0.303	0.328*	0.334*	0.449**	0.474**	− 0.092	–	–	–	–
*Sleep outcomes*													
AHI	0.227	− 0.106	0.402*	0.134	0.346	0.212	0.066	0.153	− 0.204	0.237	0.281	− 0.075	− 0.073
N1	0.008	− 0.044	0.301	− 0.132	0.033	0.109	− 0.056	− 0.035	− 0.044	− 0.115	− 0.088	− 0.118	− 0.031
N2	0.106	− 0.071	− 0.108	0.238	0.192	0.027	0.189	0.249	0.084	− 0.009	0.295	0.078	0.064
N3	0.044	0.185	− 0.188	0.217	0.001	− 0.023	0.044	0.099	0.070	− 0.103	0.084	0.050	− 0.007
Paradoxical sleep	0.014	− 0.415*	0.025	− 0.095	0.190	− 0.152	− 0.319	− 0.076	0.084	− 0.230	− 0.121	− 0.279	− 0.188
Arousal (N)	0.392*	0.167	0.299	0.099	0.118	0.369*	0.204	0.204	− 0.098	0.054	0.202	0.058	− 0.024
Arousal (N/h)	0.289	0.099	0.321	0.022	0.035	0.172	0.148	0.106	− 0.089	0.019	0.110	0.010	− 0.080
Saturation < 90%	0.250	0.097	0.354*	0.019	0.088	0.265	0.063	0.084	− 0.041	− 0.071	0.062	0.003	− 0.019
Epworth	0.205	− 0.006	0.229	0.165	− 0.014	0.115	0.210	0.141	0.040	0.081	− 0.009	0.022	− 0.237
Pichot	0.208	0.157	0.060	− 0.146	0.112	0.177	− 0.014	0.055	0.454**	0.188	0.079	− 0.039	− 0.108

Tonsil score was the score of tongue tonsil thickening

*AHI* apnea–hypopnea index, *BMI* body mass index, *RSA* reflux sign assessment, *RSS-12* reflux symptom score-12

*p < 0.05; **p < 0.01; ***p < 0.001

The associations between reflux and sleep findings are described in Table 4. Focusing on reflux signs, the tongue-base hypertrophy severity was significantly associated with the BMI (r_s_ = 0.419; *p* = 0.010); the number of micro arousals (r_s_ = 0.369; *p* = 0.027); the supine percent of pH < 6.5 (r_s_ = 0.358; *p* = 0.030); the upright percent of pH < 6.5 (r_s_ = 0.334; *p* = 0.044); the Ryan score (r_s_ = 0.382; *p* = 0.020); the morning (r_s_ = 0.373; *p* = 0.030) and bedtime pepsin saliva measurements (r_s_ = 0.365; *p* = 0.037).

Concerning associations between reflux and sleep outcomes, the analysis reported a significant positive association between the level of pepsin at bedtime and the AHI (r_s_ = 0.402; *p* = 0.017). There was a negative association between the duration of the paradoxical sleep phase and the level of pepsin in the morning (r_s_ = − 0.415; *p* = 0.013). The level of bedtime pepsin was associated with the duration of the sleep time with a saturation < 90% (r_s_ = 0.354; *p* = 0.040). There was no significant association between OSA and the GI endoscopy findings.

## Discussion

The association between gastroesophageal reflux disease, laryngopharyngeal reflux and sleep apnea remains a controversial topic. Many studies reported a high prevalence of reflux in OSA patients [7, 9, 19], but the underlying pathophysiological mechanisms of this association are still unknown.

In the present study, patients underwent oropharyngeal pH-monitoring, pepsin saliva measurements and polysomnography at the same time. The primary finding was the observation of a high prevalence of LPR in OSA patients at the oropharyngeal pH-monitoring (93%), which corroborates the findings of the literature. Indeed, the prevalence of reflux in OSA patients ranges from 38.9% to 100% according to studies [19–23]. The LPR prevalence was estimated to 10% to 30% of general population or population consulting in otolaryngology department [24–26]. In our study, most reflux events were nonacid (pH < 6.5), which corroborates the findings of Wang et al. [27] who reported that OSA patients treated with CPAP had nonacid LPR at the oropharyngeal pH-monitoring.

Interestingly, we observed that most oropharyngeal reflux events occurred supine and nighttime. This supine/nighttime profile of LPR does not corroborate the typical LPR profile of patients without OSA at the oropharyngeal pH-monitoring [28] or hypopharyngeal impedance-pH monitoring (HEMII-pH) [13, 19, 29]. Indeed, LPR patients have commonly upright and daytime pharyngeal reflux events, while the supine/nighttime events represent a low proportion of the 24-h reflux events [13, 19, 28, 29]. The higher BMI of OSA patients and the related higher risk of GERD [30], which is commonly associated with supine events, may support our observation.

Our analysis reported significant associations between Ryan score, reflux time at pH < 6.5 in supine position, pepsin measurements and high scores at the RSA, especially tongue-base hypertrophy scores. The association between fibroscopic finding severity, pepsin saliva level and oropharyngeal pH-monitoring may support the key role of pepsin in the development of laryngopharyngeal inflammatory reaction of the mucosa and the related-edema in OSA patients. Thus, it seems conceivable that the high proportion of reflux time and the related deposit of pepsin lead to an increase of the tongue-base edema and a worsening of the apnea findings. The positive association between the severity of the tongue-base hypertrophy and the occurrence of the micro-arousal events at the polysomnography may support the role of reflux, mucosa edema and OSAS. Interestingly, the influence of reflux on the tongue-base edema of OSA patients was furthermore supported in the study of Sung et al. who observed high reflux finding scores in OSA patients [31]. The association between pH-monitoring and sleep features at the polysomnography was investigated in many studies [20–22, 32–34], which reported controversial findings. Patients with severe OSA reported significant higher number of nocturnal proximal or distal esophageal events compared with patients with mild OSA in few studies [20, 34], while some authors did not find significant associations between reflux and OSA findings [21, 22]. The inconsistencies between studies are probably due to methodological differences in the evaluation of reflux events, which was carried out with esophageal dual- or triple-probe pH monitoring [22, 33], or only esophageal distal probe [20, 30]. Most of these pH-study instruments do not consider weakly acid or nonacid pharyngeal events. To date, it is known that a significant number of proximal esophageal reflux events do not reach pharyngeal region, which supports the need to use device with pharyngeal sensors, such as HEMII-pH or oropharyngeal pH-monitoring [35].

The primary limitation of this study were the low number of patients and the lack of control group. Because oropharyngeal pH-monitoring is costly and inconvenient, it was difficult to convince patients to have polysomnography and pH-monitoring at the same time. The investigation of the LPR prevalence in healthy individuals and the correlation analysis between fiberoptic findings and pH testing features should have improved the quality of the present study. Another potential limitation was the lack of use of HEMII-pH, which may provide important findings on esophageal reflux and GERD. In addition, HEMII-pH is currently considered as the most reliable device in the LPR diagnosis but a recent investigation supported the reliability of oropharyngeal pH-monitoring in patients who underwent HEMII-pH and oropharyngeal pH-monitoring in the same 24-h period [36]. From a clinical standpoint, the anatomical features of patients were investigated with RSA. The addition of Friedmann or Mallampati scores should improve the clinical checkup in our population. Moreover, some confounding factors might have biased the clinical evaluations (e.g. pollution).

The primary strengths of the study were the realization of oropharyngeal pH-monitoring and polysomnography in the same period and the measurement of pepsin saliva concentration at two important times, highlighting the pepsin deposit in the upright/daytime period and the supine/nighttime period of the 24-h pH-monitoring period. To the best of our knowledge, this is the first study using such approach combining pH-monitoring, pepsin measurement and polysomnography at the same time.

## Conclusion

OSA patients reported high prevalence of reflux events at the oropharyngeal pH-monitoring and high level of pepsin saliva measurement compared to normative data. The findings of the present study may support a potential relationship between oropharyngeal reflux events, pepsin saliva level, pharyngeal signs and micro-arousals but these findings need to the confirmed in future controlled studies.

## Abbreviations

HEMII-pH: Hypopharyngeal-esophageal multichannel intraluminal impedance-pH monitoring
LPR: Laryngopharyngeal reflux.
OSAS: Obstructive-sleep apnea syndrome
